# Are patients with lichen planus really prone to urolithiasis? Lichen planus and urolithiasis

**DOI:** 10.1590/S1677-5538.IBJU.2015.0364

**Published:** 2016

**Authors:** Ural Oguz, Zennure Takci, Isil Deniz Oguz, Berkan Resorlu, Ilknur Balta, Ali Unsal

**Affiliations:** 1 Department of Urology, School of Medicine, University Giresun, Giresun, Turkey;; 2 Department of Dermatology, School of Medicine, University Gaziosmanpasa, Tokat, Turkey;; 3 Department of Dermatology, Prof. Dr. A. Ilhan Ozdemir State Hospital, Giresun, Turkey;; 4 Department of Urology, School of Medicine, University Onsekiz Mart, Canakkale, Turkey;; 5 Department of Dermatology, Kecioren Training and Research Hospital, Ankara, Turkey;; 6 Department of Urology, School of Medicine, University Gazi, Ankara, Turkey

**Keywords:** Lichen Planus, Oral, Urolithiasis, Skin Diseases, Papulosquamous, Metabolic Diseases

## Abstract

**Purpose:**

to investigate whether patients with lichen planus (LP) are really prone to urolithiasis or not.

**Patients and Methods:**

We performed a prospective analysis of 40 patients diagnosed with lichen planus (LP) (group I), and 40 volunteers did not have LP before (group II). Participants were all checked for urolithiasis by radiological investigations. Blood samples were analyzed for biochemistry parameters including calcium and uric acid. 24-h urine samples were analyzed to investigate oxalate, citrate calcium, uric acid, magnesium, sodium and creatinine.

**Results:**

Men/women ratio and mean age were similar between group I and II (p>0.05). A presence or history of urolithiasis was detected in 8 (20%) and 2 (%5) patients in group I and II, respectively (p<0.05). Hypocitraturia was the most common anomaly with 35% (n:14) in group I. The rate of hypocitraturia in group II was 12.5% (n:5) and the difference was statistically significantly different (p=0.036). In group I, hyperuricosuria and hyperoxaluria followed with rates of 27.5% (n:11) and 25% (n:10), respectively. The rate of hyperuricosuria and hyperoxaluria were both 5% (n:2) in group II and the differences were significant (p<0.05). Hyperuricemia was another important finding in the patients with LP. It was detected in 13 (32.5%) patients in group I and in 1 (2.5%) participant in group II (p=0.001).

**Conclusion:**

According to our results, metabolic disorders of urolithiasis were highly detected in the patients with LP. However, similar to the etiology of LP, the exact reasons for these metabolic abnormalities in LP remain a mystery.

## INTRODUCTION

Lichen planus (LP) is a common papulosquamous inflammatory skin disease, the etiology of which is unclear. It is estimated that the disease affects 0.5% to 2.0% of the general population. The disease is more common in females than males and is mostly detected in middle-aged patients 30-60 years of age (1). The cutaneous lesions are flat-topped, polygonal, shiny pinkish-purple papules and plaques and are faintly erythematous to violaceous. The disease is defined as unpredictable and continues approximately for 1 to 2 years. However, it is a chronic disease. It may present with exacerbations or be quiescenct for many years. The duration and response to therapy varies according to the subtype of the LP (2).

Importantly, some diseases, such as hepatitis, anxiety, hypertension, diabetes mellitus or urolithiasis, can accompany LP (3-8). According to the results of a limited number of articles, urolithiasis is a common disease in patients with LP, although its cause and etiology are unknown. It was shown that some metabolic disorders associated with urolithiasis are more common in LP. However, on the other hand, it is not known if the LP is the causative factor or result of urolithiasis. Because there is limited literature about the association of urolithiasis and LP, we aimed to investigate if patients with LP are really prone to urolithiasis and if urolithiasis is a concern in this population.

## MATERIAL AND METHODS

### Patients

After obtaining approval of the Institutional Ethics Committee, we performed a prospective analysis of 40 patients diagnosed with LP and 40 participants without any prior skin disease such as LP. We created two groups for our study. Group-I was 40 patients with LP. Group-II was the control group of 40 volunteers without LP.

Patients with anatomic predisposing factors to urolithiasis, such as a horseshoe kidney, polycystic renal disease, malrotated or ectopic kidney, ureteropelvic junction obstruction were excluded. Patients with proteinuria, glomerular or tubular renal disease, chronic renal insufficiency and uncontrolled diabetes mellitus were also excluded. Volunteers in the control group were recruited from the patients who visited the urology or dermatology outpatient clinic for any reason and did not exhibit LP or any skin disease at the time of enrollment or before.

All the patients gave a detailed history including family history of urolithiasis, prior urolithiasis history, medications, additional comorbidities and dietary habits. The patients were all routinely evaluated using a plain abdominal X-ray and ultrasonography. Computed tomography (CT) or intravenous urography was used for patients with non-opaque stones. LP was easily diagnosed by clinically visualizing the lesions in 35 (87.5%) patients. Five (12.5%) patients required punch biopsies for the diagnosis.

One (2.5%) individual in the control group had a history of urolithiasis, and 1 (2.5%) individual had a kidney stone detected that was 2mm in diameter. The 24-h urine analysis results of the patient and control groups were compared in the present study. Spot urine samples were analyzed to detect an infection. A metabolic evaluation was postponed when a urinary system infection or hematuria was detected. Blood samples were analyzed for biochemistry parameters including calcium and uric acid. Oxalate, citrate, calcium, uric acid, magnesium, sodium and creatinine were analyzed in the 24-h urine samples.

The normal constituent values of a 24-h sample are <300mg/day for calcium; <750mg/day for uric acid; <44mg/day (man) and <31mg/day (woman) for oxalate; >320mg/day for citrate; <73mg/day for magnesium; <220mg/day for sodium; between 600-1600mg/day for creatinine; and >1200mL/day for urine volume.

Before the metabolic evaluation, participants were asked not to change their dietary habits. Medications that could affect the urinary excretion rates of stone forming substances were stopped at least 1 week prior to metabolic evaluation.

### Statistical analysis

All statistical analyses were performed using SPSS, version 20.0. Statistical significance was considered at p<0.05. As a supplementary statistic, frequency (percent) for the variables obtained by counting and mean±standard deviation and median (minimum and maximum) values for the variables obtained by measuring were used. A Chi-square analysis was used for the variables obtained by counting.

## RESULTS

The men/women ratio was approximately 3/2 in both groups I and II (p>0.05). The mean ages were 46.2 years (22-77 years) and 40.8 years (21-71 years) for groups I and II, respectively (p>0.05).

Dietary habbits that could affect the results (vegetarian, meat-based, too salty, etc.) were not detected in the individuals. The presence or history of urolithiasis was detected in 8 (20%) patients with LP. A renal calculus smaller than 3mm was detected in 2 (5%) of the patients at the time of LP presentation. Six (15%) of the patients had a history of previous spontaneous calculus passage. In the control group (group-II), 1 (2.5%) individual had a history of urolithiasis, and a kidney stone 2mm in diameter was detected in 1 (2.5%) individual. We could not assess any results of the patient’s calculus analyses.

A family history of urolithiasis was highly detected in group-I. Thirteen (32.5%) individuals in group-I and 3 (7.5%) individuals in group-II had a family history of urolithiasis (p<0.05).

The additional comorbidities detected in groups I and II were hypertension (4 versus 4), diabetes mellitus (4 versus 2) and malignancy (0 versus 1). One patient in group-II had a history of a partial nephrectomy due to an exophytic right renal mass 2cm in diameter approximately 10 years ago. After a curative treatment, no recurrence was detected, and his renal function was completely normal (Table-1).

**Table 1:** The distribution of comorbid conditions, family history of urolithiasis and prior stone history.

			Group I		Group II		All		Chi-square test	

			n	%	n	%	n	%	Chi-square	p
**Family history of urolithiasis**		absent	27	67.5	37	92.5	64	80.0	6.328	**0.012**
		existence	13	32.5	3	7.5	16	20.0		
		all	40	100.0	40	100.0	80	100.0		
**Prior stone history**		absent	34	85.0	39	97.5	73	91.2	Fisher’s Exact	0.108
		existence	6	15.0	1	2.5	7	8.8		
		all	40	100.0	40	100.0	80	100.0		
**The presence or history of urolithiasis**		absent	32	80.0	38	95.0	70	87.5	2.857	0.091
		existence	8	20.0	2	5.0	10	12.5		
		all	40	100.0	40	100.0	80	100.0		
**Comorbidities**	**Hypertension**	absent	36	90.0	36	90.0	72	90.0	Fisher’s Exact	1
		existence	4	10.0	4	10.0	8	10.0		
		all	40	100.0	40	100.0	76	100.0		
	**Diabetes Mellitus**	absent	36	90.0	38	95.0	74	92.5	Fisher’s Exact	0.675
		existence	4	10.0	2	5.0	6	7.5		
		all	40	100.0	40	100.0	76	100.0		
	**Malignancy**	absent	40	100.0	39	97.5	79	98.8	Fisher’s Exact	1
		existence	0	0.0	1	2.5	1	1.2		
		all	40	100.0	40	100.0	80	100.0		

Hypocitraturia was the most common anomaly with 35% (n: 14) in group-I. The rate of hypocitraturia in group-II was 12.5% (n: 5). The difference between the two groups was statistically significantly different (p=0.036) (Table-2).

**Table 2:** The distribution of metabolic analysis results.

		Group I		Group II		All		Chi-square test	

		n	%	n	%	n	%	Chi-square	p
**Hypercalciuria**	absent	34	85	33	82.5	67	83.75	0.000	1.000
	existence	6	15	7	17.5	13	16.25		
	all	40	100.0	40	100.0	80	100.0		
**Hyperuricosuria**	absent	29	72.5	38	95	67	83.75	5.878	0.015
	existence	11	27.5	2	5	13	16.25		
	all	40	100.0	40	100.0	80	100.0		
**Hyperoxaluria**	absent	30	75	38	95	68	85	4.804	0.028
	existence	10	25	2	5	12	15		
	all	40	100.0	40	100.0	80	100.0		
**Hypocitraturia**	absent	26	65	35	87.5	61	76.25	4.418	0.036
	existence	14	35	5	12.5	19	23.75		
	all	40	100.0	40	100.0	76	100.0		
**Hypomagnesiuria**	absent	29	72.5	34	85	63	78.75	1.195	0.274
	existence	11	27.5	6	15	17	21.25		
	all	40	100.0	40	100.0	76	100.0		
**Hypernatriuria**	absent	28	70	30	75	58	72.5	0.063	0.802
	existence	12	30	10	25	22	27.5		
	all	40	100.0	40	100.0	80	100.0		
**Low urine volume**	absent	35	87.5	34	85	69	86.25	0.000	1.000
	existence	5	12.5	6	15	11	13.75		
	all	40	100.0	40	100.0	80	100.0		
**Hypercalcemia**	absent	37	92.5	39	97.5	76	95	Fisher's Exact	0.615
	existence	3	7.5	1	2.5	4	5		
	all	40	100.0	40	100.0	80	100.0		
**Hyperuricemia**	absent	27	67.5	39	97.5	66	82.5	10.476	0.001
	existence	13	32.5	1	2.5	14	17.5		
	all	40	100	40	100	80	100		

In group-I hyperuricosuria and hyperoxaluria followed with rates of 27.5% (n: 11) and 25% (n: 10), respectively. The rates of hyperuricosuria and hyperoxaluria were both 5% (n: 2) in group-II. The differences between the groups were significant (p<0.05) (Table-2).

Hypercalciuria was detected in 6 (15%) and 7 (17.5%), hypernatriuria in 12 (30%) and 10 (25%), hypomagnesiuria in 11 (27.5%) and 6 (15%), and low urine volume in 5 (12.5%) and 6 (15%) of the individuals in groups I and II, respectively. These findings were statistically similar between the patient and control groups (p>0.05).

Hyperuricemia was another important finding in patients with LP. It was detected in 13 (32.5%) patients in group I and in 1 (2.5%) participant in group-II (p=0.001). Three (7.5%) patient’s in group-I and 1 (2.5%) participant in group-II had hypercalcemia, which was not significant (p>0.05). The metabolic analysis results of the groups are detailed in Table-2.

## DISCUSSION

Lichen planus (LP) is an inflammatory disease that can occur on the skin, nails, hair or mucosal membranes. The incidence of the disease is unclear, but it is thought that approximately 1% of the general population are effected by the disease (9, 10). The cutaneous lesions are flat-topped, polygonal and shiny pinkish-purple papules and plaques. Reticulated whitish punctate networks called Wickham striae that can typically be seen over most of the papules is a characteristic finding of the disease (9, 11). While LP can be diagnosed by easily visualizing the lesions, a punch biopsy can be required to diagnose LP in some patients.

Although the etiology is unknown, immunologic mechanisms are known to be responsible for the formation of the lesions. When the phenotype of inflammatory infiltration was investigated, increased CD4^+^ and particularly CD8^+^ T-cells were observed within the epithelium and around the damaged basal keratinocytes. Following the antigen recognition that activates the T-cells, cytokines and chemokines, such as interferon-γ, tumor necrosis factor-α, transforming growth factor-β1, interleukin-2, interleukin-4 and interleukin-10, are released. Severity of the disease is based on the balance between the two extremes of lymphocytic activation and down regulation (9, 12).

LP can accompany some different illnesses. Gavic et al. (8) showed that LP is associated with anxiety and depression. However, in contrast, Hirota et al. (13) presented that there was no correlation between anxiety and LP. Some articles have investigated the coexistence of LP and hepatitis, hypertension, diabetes mellitus and urolithiasis. However, the relationship of these diseases were not identified (3-7).

Urolithiasis is a common disease that effects approximately 11% of the adult population. To date, a limited number of studies have evaluated the association of urolithiasis and LP (6, 14-16). Halevy et al. reported the coexistence of LP and urolithiasis for the first time in 1983 (15) From medical records and anamnesis, they found that 14.6% of 130 patients with LP had a history of urolithiasis. They claimed that this incidence was higher than the population of their community. In 1990, Halevy and Feuerman evaluated 42 patients with lichen planus with a biochemical analysis and 24-h urine results (16). At least one of the abnormalities, including hyperuricemia, hyperuricosuria or hypercalciuria, was detected in 9 (21%) patients. According to their results, they concluded that there could be involvement of metabolic disorders in LP. The last article on this topic was published by Kumar et al. in 1999 (6) and included 75 patients with LP and 62 healthy individuals. Nine (12%) of those patients had a history of urolithiasis. They checked the patients for urinary system stones with ultrasonography. Three (4%) of the patients had kidney stones detected at the time of presentation. They used blood samples to analyze biochemical parameters, including calcium and uric acid, and a 24 hours urine collection to analyze calcium, uric acid, phosphorus, urea and creatinine. Unlike previous literature (16), their results were similar between the patient and control groups. The authors concluded that the only noticeable result of their study was that the serum uric acid and urine calcium levels and prior history of urolithiasis were significantly higher in the patients with LP compared to the control group (6). We think an important limitation of their study was that they did not investigate other lithogenic factors, such as urine oxalate, citrate, magnesium or sodium levels, which can be considered important risk factors for urolithiasis (17).

Our results demonstrated a 20% prevalence of urolithiasis in patients with LP, which can be considered similar to previous studies (6, 15). In 5% of the patients, a renal calculus at the time of presentation was detected. Fifteen percent of the patients had a previous history of spontaneous calculus passage. In the control group, 5% of the participants had urolithiasis or a history of urolithiasis. Although it seems to be high in the patient group, it was not statistically significant, perhaps due to the low number of the patients (p: 0.09). None of the patients had the results of their calculus analyzed.

Hyperuricemia was detected in 32.5%, hyperuricosuria in 27.5%, hyperoxaluria in 25%, hypocitraturia in 35% of the patients. These findings were significantly higher than the control group (p<0.05).

We hypothesize that the turn-over around the lesions could be mainly associated with the increased rate of hyperuricosuria and hyperuricemia. The high incidence of hyperoxaluria and hypocitraturia was revealed for the first time by our study. However, it is unclear why these metabolic abnormalities occur in LP. Although patients with LP seem to be prone to urolithiasis due to the metabolic disorders mentioned above and have a high incidence of a history of urolithiasis, none of the patients had a history of staghorn calculus or stone surgeries. The 3mm diameter calculuses diagnosed at the time of presentation in 2 patients were clinically insignificant. On the other hand, a history of urolithiasis was mostly experienced before the LP disease, which is similar to the literature (6, 15).

In addition, our results demonstrated a high rate of family history of urolithiasis in patients with LP (32.5% group-I versus 7.5% group-II). This finding led us to uncertainty about which was the reason and which was the result.

According to our results, the metabolic disorders of urolithiasis were highly detected in the patients with LP. However, the main reason for the metabolic abnormalities remains a mystery in LP, similar to the etiology of LP.

We acknowledge that CT was not routinely used to avoid radiation exposure when detecting the urinary system stones and was an important limitation within our study. However, the study was designed as a prospective study. The patients were all evaluated using plain abdominal X-ray and ultrasonography to avoid radiation exposure. A CT was required only in the patients with a history of non-opaque stones. In addition, we did not routinely use punch biopsies to diagnose the disease. LP is diagnosed by visualizing typical lesions, and a punch biopsy is required only in patient’s with a suspicious diagnosis. The low number of patients could also be considered another limitation of the study. Despite these shortcomings, this is an important study because there are limited numbers of studies about this topic.

## CONCLUSIONS

According to our results, metabolic disorders of urolithiasis were highly detected in the patient’s with LP. However, there were no mortality or morbidity consequences of the urolithiasis disease to the patient’s. Similar to the etiology of LP, the exact reasons for these metabolic abnormalities in LP remain a mystery. Further studies are necessary to clarify this mystery.
